# Quantitative Analysis of the Bacteria in Blepharitis With Demodex Infestation

**DOI:** 10.3389/fmicb.2018.01719

**Published:** 2018-07-31

**Authors:** Minyi Zhu, Chao Cheng, Haisu Yi, Liping Lin, Kaili Wu

**Affiliations:** Zhongshan Ophthalmic Center, State Key Laboratory of Ophthalmology, The Key Lab of Ophthalmology and Visual Science of Guangdong, Sun Yat-sen University, Guangzhou, China

**Keywords:** bacteria, demodex mites, blepharitis, eyelashes, MALDI-TOF-MS

## Abstract

Bacterial infection or Demodex infestation has been reported to contribute to chronic blepharitis. The association between Demodex mites and bacterial flora in this disease remains to be elucidated. Fifty-six consecutive patients diagnosed with chronic blepharitis and 46 healthy volunteers were recruited for this study. Using specimens of three epilated eyelashes and lid margin swabs, Demodex were identified microscopically and bacteria were determined by cultures, followed by colony counting and mass spectrometry. We found 191 Demodex mites, 161 D. folliculorum and 30 D. brevis, in 45 patients. Correspondingly, 101 Demodex, 63 D. folliculorum and 38 D. brevis, were found in 21 controls (*p* < 0.05, both). Bacterial culture-positivity was obtained in samples (eyelashes, lid margins, or both) from 54 patients and in eyelashes from 37 controls. The total colony counts and the incidences and colony counts of *Propinibacterium acnes* and *Staphylococcus aureus* from patients’ eyelashes were significantly higher than that of the controls. Furthermore, bacterial colony counts in blepharitis patients’ eyelashes with D. folliculorum were higher than that of controls with D. folliculorum (*p* < 0.01). Similarly, *P. acnes* colonies increased significantly in patients’ eyelashes with D. folliculorum (*p* < 0.05). These results suggest that D. folliculorum and *P. acnes* have a role in the occurrence of the chronic blepharitis. Further studies are required to reveal the relationship between these two organisms in blepharitis.

## Introduction

Blepharitis is a common eye condition characterized by inflammation of the eyelid margins, with a sensation of burning, stinging, and itching and clinical evidence of scales around the eyelashes, lid margin erythema, telangiectasia, thickening, irregularity of the eyelid margins and conjunctival hyperemia. Very little is known of the incidence or prevalence of this disorder. In one study, it was reported that blepharitis was found in 37% of patients in the ophthalmologist’s clinical practice in the United States (Lemp and Nichols, 2009). Another study on 1148 consecutive patients with ocular discomfort or irritation reported anterior blepharitis in 12% and posterior blepharitis in 24% (Venturino et al., 2003). Multiple diseases and conditions, such as allergies, rosacea, clogged oil glands and/or meibomian gland dysfunction, as well as bacteria, viral, and Demodex mite infections, were considered to be the causes of blepharitis (Jackson, 2008; Liu et al., 2010).

Blepharitis caused by various bacterial infection has been receiving considerable clinical attention. The most common bacteria in chronic blepharitis include *Staphylococci*, *Corynebacterium*, and *Propronibacterium* species (Dougherty and McCulley, 1984; Teweldemedhin et al., 2017). In one study of the microbiota of blepharitis comparing 332 consecutive patients with chronic blepharitis to 160 control patients, Groden et al. (1991) reported that the most commonly isolated organisms from lids with blepharitis were *Staphylococcus epidermidis* (95.8%), *Propinibacterium acnes* (92.8%), *Corynebacterium* spp. (76.8%), *Acinetobacter* spp. (11.4%), and *Staphylococcus aureus* (10.5%) based on cultivable approaches. Similarly, by analyzing the 16S rRNA microbiome from lashes and tears of people with and without blepharitis, Lee et al. (2012) found that the microbiome mainly consisted of five genera (i.e., *Propionibacterium*, *Staphylococcus*, *Streptophyta*, *Corynebacterium*, and *Enhydrobacter*), but the differences between people with and without blepharitis, especially for the microbiome, were not statistically significant. Thus, antibiotics are widely used for the treatment of blepharitis in clinical practice (Pflugfelder et al., 2014).

Two types of Demodex mites, Demodex folliculorum (D. folliculorum), and Demodex brevis (D. brevis), were identified in humans and were reported to be associated with eye disorders, such as blepharitis, dry eye, conjunctivitis, and corneal lesions (Gao et al., 2007; Liang et al., 2014). The prevalence of mite infestation increases with age, and ∼58% of individuals with mites will have evidence of chronic blepharitis (Elston, 2010). Dadaci reported that direct microscopic examination revealed Demodex mites in 42.1% of the blepharitis group (Dadaci et al., 2015). In the eyelids, D. folliculorum are frequently found in hair follicles, while D. brevis has been thought to live in sebaceous glands (meibomian and Zeiss glands) (Liu et al., 2010). Therefore, D. folliculorum was supposed to cause chronic anterior blepharitis, while D. brevis has been considered to cause posterior blepharitis (Cheng et al., 2015). Meanwhile, there have been some studies that have suggested that metronidazole and tea tree oil are effective choices for the treatment of demodex infestation in blepharitis (Cheng et al., 2015; Akcinar et al., 2016). However, most people who have these obligate mites are healthy and there is no rigorous evidence demonstrating that Demodex mite infestation promotes bacterial infection in blepharitis (Clifford and Fulk, 1990; Bezza Benkaouha et al., 2015). Exploring the reason that mites contribute to blepharitis will be beneficial to therapy. Recently, people have been making significant efforts to explore the important relationship between the presence of Demodex and blepharitis (Kosik-Bogacka et al., 2012; Bhandari and Reddy, 2014; Cheng et al., 2015). The Demodex mite was supposed to act as a carrier of *Bacillus oleronius* that was found inside the parasite, which may function as a co-pathogen in the pathogenesis of blepharitis (Lacey et al., 2007; Szkaradkiewicz et al., 2012).

The purpose of the present study was to investigate the bacterial flora in eyelid margins and hair follicles infested with Demodex in chronic blepharitis as well as to quantify the mite and bacteria loads compared with normal individuals. To the best of our knowledge, there is no previous report of a quantitative analysis of the bacteria between the pulled eyelashes and lid margin in chronic blepharitis with Demodex infestation. In our opinion, the discovery of specific bacteria associated with demodex infestation in blepharitis may contribute to improved blepharitis treatment.

## Materials and Methods

### Patients

All patients were referred to the Zhongshan Ophthalmic Center between May 2016 and June 2017 and had a primary diagnosis of chronic blepharitis. A total of 56 patients were consecutively included in the study. Meanwhile, 46 normal subjects without ocular or systemic disorders and have not used topical or systemic antibiotics within 2 weeks, matched by age and gender, were recruited for the control group. Study subjects were examined using a slit-lamp biomicroscope to screen for the presence of ocular signs of blepharitis. The diagnosis was based on symptoms and the ocular findings, as described by AAO Blepharitis: Preferred Practice Pattern Guideline 2013^1^. This study was approved by the Human Research Ethics Committee of Zhongshan Ophthalmic Center, and all subjects provided written informed consent.

### Demodex Examination

The sampling of eyelashes for Demodex examination was conducted as we previously reported with modification (Li et al., 2010; Liang et al., 2014). Briefly, the Demodexes in patients with a cylindrical sleeve, crusting, scurf or matting were chosen. Three eyelashes, isolated and taken from the upper lid of a patient or a healthy subject by sterile forceps, were placed on a pre-sterilized, disposable glass slide. A drop of sterile saline was mounted on the Demodexes. Under the microscope, a positive result involved the presence of at least one adult, larva, protonymph, or nymph stage of D. folliculorum or D. brevis. In parallel, the number and species of Demodex were counted. The process of Demodex examination was performed under sterile conditions. For reviewing the results, all eyelid conditions and findings on slides were photographed with cameras built into microscopes. After checking and recording the Demodex, the sample (eyelashes ± mites in saline) on the slide was immediately inoculated onto plates using FLOQSwabs^TM^ (Copan Italia, Brescia, Italy). An ophthalmic surgeon (MY Zhu) collected eyelash specimens from all subjects.

### Bacterial Culture and Identification

A complete microbiologic evaluation of cultures from the eyelid margins and eyelashes was performed. All protocols were conducted as we previously reported with modification (Wang et al., 2015). Briefly, by using a moistened Copan flocked swab with sterile saline, the lid margin samples were obtained by wiping twice along the upper lid margin from the angle to the punctum. Then, the swabs were seeded onto a culture plate (chocolate agar + polyViteX, bioMérieux, Shanghai, China). In addition, the residues on glass slides, which were used for the Demodex examination, were inoculated onto the plate by the Copan flocked swab. Both aerobes and anaerobes were routinely cultured separately at 35° for up to 7 days. The grown colonies were macroscopically evaluated and recorded.

Identification of the bacterial colony was performed using MALDI-TOF-MS (VITEK MS, bioMérieux, France) according to what we reported previously (Song et al., 2017). Briefly, a single colony of isolates grown on a plate was selected, transferred to a target slide, and overlaid with CHCA matrix solution (bioMérieux). Each sample that passed the auto-quality control was achieved. *E. coli* ATCC 8739 served as a calibrated control in the acquisition. For multiple types of colonies on a plate, a representative colony from each type was separately identified by MALDI-TOF-MS and noted.

### Statistical Analysis

A Chi-square test was used for frequency data analysis and the Mann–Whitney test for attributes data analysis. All statistics were performed with SPSS 20.0, and the level of significance for all tests was adopted as 0.05.

## Results

### Demodex Findings

A total of 56 patients (32 females and 24 males, 34.3 ± 16.3 years) and 46 normal individuals (29 females and 17 males, 40.4 ± 19.2 years), matched by age and gender, were consecutively recruited for this study. Demodex was found in 80.36% (45/56) of patients with blepharitis and 45.65% (21/46) of controls (*p* < 0.01). The average number of Demodex mites on three eyelashes per patient was 3.41 ± 3.86, while the average was 2.20 ± 3.66 in control subjects (*p* < 0.01). There were 191 Demodex (D. folliculorum: 161, D. brevis: 30) found in blepharitis patients. Additionally, 101 Demodex (D. folliculorum: 63, D. brevis: 38) were found in controls. For the incidence and counts of D. folliculorum, the difference was significant between blepharitis patients and controls (*p* < 0.01), while no significant difference was observed for D. brevis (*p* > 0.05; **Table 1**).

**Table 1:** Prevalence of two Demodex mites in blepharitis patients and controls.

	Blepharitis patients (*n* = 56) (%)		Controls (*n* = 46) (%)	
		
	Positive subjects	Total demodex	Positive subjects	Total demodex
Demodex	45 (80.36)^∗^	191	21 (45.65)	101
D. folliculorum	43 (76.79)^∗^	161 (84.29)^∗^	19 (41.30)	63 (62.38)
D. brevis	17 (30.36)	30 (15.71)	15 (32.61)	38 (37.62)


*^∗^*P* < 0.01 (patient vs. control).*

### Bacteriological Findings

Culture of patient samples revealed culture-positivity in 54 samples of eyelashes (96.55%) and 54 samples of lid margins (96.55%) from 56 blepharitis patients and in 37 of 46 (80.43%) eyelashes from healthy individuals. There were 27 species of 16 genera of bacteria in blepharitis samples, while only 16 species of 11 genera, in healthy individuals. **Table 2** displays major bacteria for three types of samples. *Propionibacterium* spp., *Staphylococcus* spp., and *Bacillus* spp. were the most frequently isolated organisms from all individuals. For anaerobic bacteria recovered from subjects, *Propionibacterium* spp., *Bacillus* spp., and *Fusobacterium* spp. were the most frequently isolated organisms. Other relatively infrequently isolated organisms included *Streptococcus* spp., *Corynebacterium* spp., *Micrococcus* spp., *Neisseria* spp., and others. In blepharitis patients, the prevalence and categories of isolates from the lid margin were similar to eyelashes. However, the bacterial distribution on eyelashes was different between the blepharitis patients and controls. There were significant differences in the culture-positive incidence of *P. acnes* and *S. aureus* between blepharitis patients and controls based on eyelash culture (**Table 2**).

**Table 2:** Culture-positive incidences of major isolates in blepharitis patients and controls.

	Blepharitis patients (*n* = 56) (%)		Control eyelashes
		
	Lid margin	Eyelash	(*n* = 46) (%)
**Aerobic**			
*Staphylococcus* spp.	36 (61.29)	39 (69.64)	27 (58.70)
*S. aureus*	22 (39.29)	27 (48.21)^∗^	12 (26.09)
*S. epidermidis*	22 (39.29)	26 (46.43)	21 (45.65)
*Streptococcus* spp.	12 (21.43)	13 (23.21)^∗∗^	2 (4.35)
*Corynebacterium* spp.	8 (14.29)	12 (21.43)	4 (8.70)
**Anaerobic**			
*Propionibacterium* spp.	42 (75.00)	42 (75.00)^∗^	13 (28.26)
*P. acnes*	41 (73.21)	42 (75.00)^∗^	13 (28.26)
*Bacillus* spp.	11 (19.64)	12 (21.43)	5 (10.87)
Total organisms	54 (96.43)	54 (96.43)^∗∗^	37 (80.43)


*^∗^*p* < 0.05 and ^∗∗^*p* < 0.01 (blepharitis vs. Control).*

**FIGURE 1:**
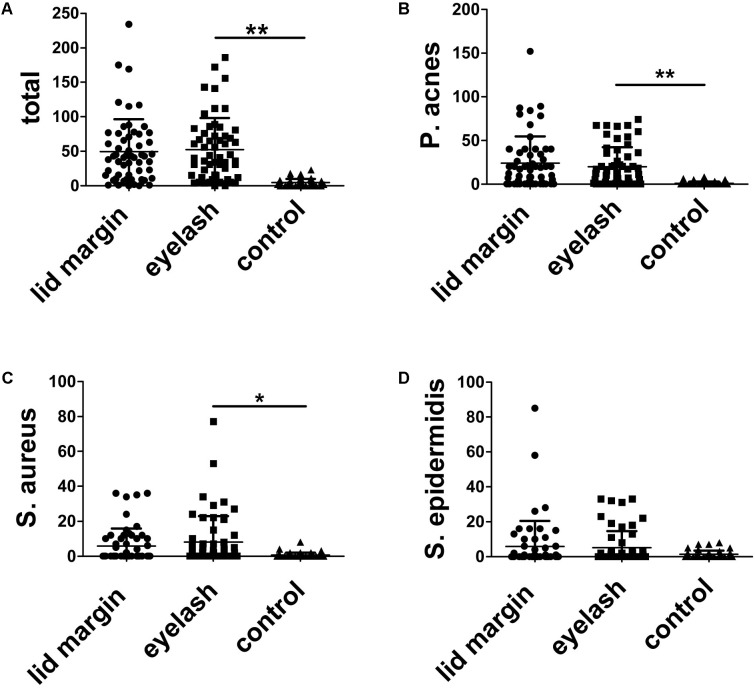
Comparison of the colonies of the total and three major bacteria among different sample groups. The counted colonies of total bacterial isolated colonies **(A)**, *Propinibacterium acnes* **(B)**, and *Staphylococcus aureus* **(C)** from the eyelashes of patients were, separately, significantly more than that of normal individuals. **(D)** As for *Staphylococcus epidermidis*, no significant differences were observed among different groups. Lid margin, lid margin of blepharitis; eyelash, eyelash of blepharitis; control, eyelash of normal individuals. ^∗^*p* < 0.05 and ^∗∗^*p* < 0.01.

We further quantified the isolates from different sample groups by counting the colonies. There were 2784 colonies (*P. acnes*, 1341; *S. aureus*, 331; and *S. epidermidis*, 335) on culture plates from the lid margins sand 2941 colonies (*P. acnes*, 1113; *S. aureus*, 454; and *S. epidermidis*, 287) for eyelashes of 56 blepharitis patients, and 212 colonies (*P. acnes*, 40; *S aureus*, 27; and *S. epidermidis*, 59) for eyelash culture of 46 controls. **Figure 1** demonstrates the colonies found in every subject for all three major bacteria in three sampling groups. The colony counts in eyelashes of blepharitis patients (52.52 colonies/person) were 10-fold higher than the control group (5.02 colonies/person) (*p* < 0.01). As expected, the colonies of both *P. acnes* and *S. aureus* were significantly higher in patient eyelashes than the normal group (*p* < 0.05, for both bacteria). However, the colony count of *S. epidermidis* in eyelashes did not show differences between patients and controls (*p* > 0.05). In addition, the bacterial loads of all three major isolates were the same between sampled eyelashes and lid margins in blepharitis patients (*p* > 0.05).

### Demodex Mites and Bacteria Coexisted in Sampled Eyelashes

As described in **Table 3**, 45 patients had positive findings for Demodex mites and all of them had positive bacterial cultures. Correspondingly, 17 out of 21 healthy individuals with Demodex had positive cultures for bacteria based on eyelashes. Meanwhile, the prevalence of *P. acne* displayed significant increases in patient eyelashes with Demodex (*p* < 0.05, vs. control).

**Table 3:** Incidences of major bacteria in the eyelashes of people with Demodex infestation.

	Eylash of patients	Eyelash of control
	*n* = 45 (%)	*n* = 21 (%)
*P. acnes*	38 (84.44)^∗^	12 (57.14)
*S. aureus*	18 (40.00)	7 (33.33)
*S. epidermidis*	23 (51.11)	10 (57.62)
Others	39 (86.67)	12 (57.14)
Total	45 (100)^∗^	17 (80.95)


**^∗^p* < 0.05 (blepharitis vs. control).*

**FIGURE 2:**
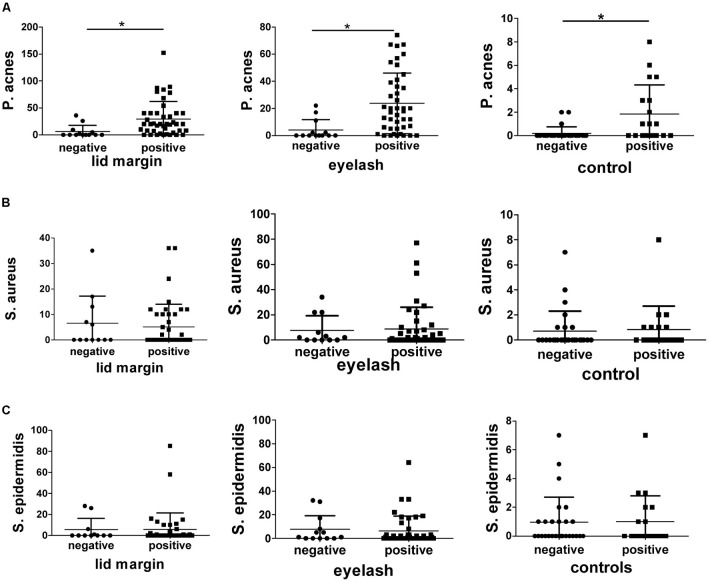
Comparisons of bacterial colonies in samples with or without D. folliculorum infestation. **(A)** Shows that the *P. acnes* colonies were significantly increased in samples with positive D. folliculorum (*p* < 0.05). By contrast, the colonies of *S. aureus* **(B)** and *S. epidermidis* **(C)** did not have differences in the presence or absence of D. folliculorum infestation in the three types of culture samples. Lid margin, lid margin of blepharitis; eyelash, eyelash of blepharitis; control, eyelash of normal individuals. ^∗^*p* < 0.05.

We further analyzed the relationship between the bacteria colonies and D. folliculorum infestation (**Figure 2**). The total bacterial loads in samples with D. folliculorum from lid margins and eyelashes of patients and eyelashes of control were 2400, 2521, and 116 colonies, respectively. For samples with D. folliculorum infestation, there were significant increases in the bacterial colonies in patients eyelashes compared to healthy people (*p* < 0.01). Similarly, the colonies of *P. acnes* increased significantly in samples with D. folliculorum infestation from patients as well as healthy individuals (*p* < 0.05). The loads of the other two bacteria, i.e., *S. aureus* and *S. epidermidis*, did not show differences if there was a D. folliculorum infestation.

## Discussion

Blepharitis can arise from the infestation of Demodex mites and/or bacterial infection. In this study, we compared the bacterial flora on eyelashes with or without Demodex mites and on the lid margin in chronic blepharitis patients. We found that there was a higher prevalence of Demodex mites and bacteria, and that there were increased colony counts in the eyelash follicles in blepharitis compared to normal subjects. More importantly, the prevalence of bacteria culture revealed a significant increase in eyelashes with Demodex infestation. The colony count of *P. acnes* in samples that were positive for D. folliculorum was significantly higher than those without D. folliculorum in all three samples from lid margins and eyelashes of patients as well as eyelashes from control individuals. In addition, the bacterial loads and categories of the lid margin were similar to those from eyelashes.

In the present study, cultures of material obtained from the three types of samples yielded similar organisms at different frequencies. The percentages of the positive cultures and bacterial loads were significantly higher for blepharitis patients compared with normal controls, which is consistent with previous studies (Willcox, 2013; Bezza Benkaouha et al., 2015). Those studies showed that the bacteria frequently found on the eyelid margin of chronic blepharitis, including *Staphylococcus*, *Propronibacterium*, and *Corynebacterium* species, were also commonly isolated from healthy eyelids. Meanwhile, early studies emphasized that *S. aureus* was the predominant isolate in blepharitis with approximately half of patients compared to <20% in normal individuals being positive cultures for *S. aureus* (Dougherty and McCulley, 1984; Jackson, 2008). Our data showed that two bacteria (i.e., *S. aureus* and *P. acnes*) displayed significantly higher positive rates on the eyelashes of patients compared to that of controls (**Figure 1** and **Table 2**). Similarly, in our previous studies on the bacterial spectra and antibiotic resistance patterns of ocular infections, we found that the most prevalent bacterial genera was *Staphylococcus* spp., of which, *S. epidermidis* accounts for 35.25% of the external ocular infection and 14.96% of the intraocular infection (Wang et al., 2015, 2016). However, *S. epidermidis*, which was nearly the most frequently isolated bacterium with the highest loads on eyelid margins of normal subjects as well as blepharitis patients, was also reported to have a significantly increased frequency in the chronic blepharitis patients at different stages (Groden et al., 1991) or those exposed to mustard gas (Karimian et al., 2011). Our present study revealed that there were no significant differences for the positive rates of *S. epidermidis* isolated among the eyelid margins of patients and eyelashes of patients and healthy subjects. These results may suggest that the bacteria, excluding *S. epidermidis*, contributed to the pathogenesis in our patients.

In addition, the incidence of *Streptococci* spp. was significantly higher in blepharitis patients as compared to that in healthy subjects (13/56 vs. 2/46, **Table 2**). Because of the small number of positive cultures, we only presented the total number of *Streptococci* spp. and did not analyze the data for different species of *Streptococci*. Furthermore, the loads of *Streptococci* spp. didn’t increase significantly in blepharitis patients (data not shown). Thus, we can’t assume that any species of *streptococci* is pathogenic in the present study.

The correlation between Demodex infestation of the eyelids and blepharitis has been investigated epidemiologically and clinically for decades. We present that there is a clearly significant increase in the mite infestation in the eyelashes of chronic blepharitis compared to healthy subjects. In addition, we found that 80.36% of blepharitis patients (vs. 45.65% of control) had Demodex mites. Similar results, i.e., a significantly high incidence and density of Demodex infestation in patients, were reported by different researchers (Zhao et al., 2012; Bhandari and Reddy, 2014). These data provided indirect support for mite-related blepharitis. Baima and Sticherling (2002) have proposed the pathological changes in the course of demodicosis before. The underlying mechanism remains to be further explored. On the other hand, there are several routine treatment options for patients with blepharitis, from maintaining eyelid hygiene to the use of antibiotics (Pflugfelder et al., 2014). Broad-spectrum antibiotics alone are widely used for the treatment of bacteria infection in blepharitis patients even when a significant demodex infestation is present. Our current results challenge these approaches. The anti-demodex therapy was emphasized by some previous studies (Cheng et al., 2015; Akcinar et al., 2016). Therefore, apart from the application of antibacterial agents, a combination of anti-demodex therapy to reduce demodex count should be considered.

Additionally, the Demodex mite was considered to be the vector for bacteria, which was demonstrated by the finding of *Bacillus oleronius* inside Demodex mites as well as the positive serum immune-reactivity to bacillus proteins in patients with ocular Demodex infestation, facial rosacea, and blepharitis (Li et al., 2010). Comparisons of our bacterial data of sampling eyelashes with or without Demodex in normal eyes and those diagnosed with blepharitis have identified new findings in the present study. We provided additional evidence to support this speculation, e.g., a high prevalence of Demodex mite infestation accompanied by higher incidences and loads of bacteria in the eyelashes as well as eyelid margin of patients with chronic blepharitis compared to healthy controls. Furthermore, we found that *P. acnes* colonies significantly increased in eyelashes with positive D. folliculorum. Bezza Benkaouha et al. (2015) conducted a similar study in a small number of subjects and did not find a significant difference for Demodex in 9 blepharitis patients (2/9, 22.2%) vs. 7 normal individuals (2/7, 28.6%). Our study further demonstrated a strong correlation between Demodex infestation, bacterial infection and chronic blepharitis.

Because Demodex is the most common obligate ectoparasite in humans, with 80% present on skin (Vollmer, 1996) and 45.65% on eyelashes (**Table 1**) in healthy people, a chronic infestation of mites in the eyelids may result in inflammation of the eyelid and ocular surface as well as secondary bacterial infection. The present study was a preliminary study that demonstrated synergic infection of bacteria and Demodex mites around the eyelashes in chronic blepharitis, but it has limitations that should be addressed. We only recruited patients with mixed blepharitis, which is usually more severe than any single type (e.g., anterior or posterior blepharitis) of this disorder, and it usually involves serious disruption of the biofilm on the eyelids (Rynerson and Perry, 2016). Accompanying Demodex infestation, the contribution of bacterial infection is more intensive in this condition. Also, it is likely that during infection/inflammation of the eyelid, the microflora of the eyelash will be “contaminated” by that of the eyelid through “pus” and other semi-liquid substances smearing the eyelashes, which will result in mixing of the microbial flora. Any approach that can avoid this kind of contamination will be valuable for the future study. In addition, the low sample size has prevented deep analyses on the subtype of bacteria and Demodex mites. These will be overcome by recruiting more patients at various statuses and by combining analyses on the molecular level in our future work.

According to this study, we conclude that in blepharitis patients, the bacterial loads sampled from eyelashes and lid margins were similar, which were abnormally different from healthy volunteers. There was a higher prevalence of Demodex mites in blepharitis patients compared to normal subjects. Our results suggested a correlation between D. folliculorum and *P. acnes* in blepharitis. Further studies are required to reveal the relationship between these two organisms in blepharitis.

## Ethics Statement

This study was carried out in accordance with the recommendations of the “Regulations for the Human Research from the Ethics Committee of Zhongshan Ophthalmic Center” with written informed consent from all subjects. All subjects gave written informed consent in accordance with the Declaration of Helsinki. The protocol was approved by the “Human Research Ethics Committee of Zhongshan Ophthalmic Center.”

## Author Contributions

MZ identified patients, collected eye specimens, observed demodex mites, cultured bacteria, took photos, and drafted the manuscript. CC cultured, quantified, identified bacteria, and analyzed data. HY handled mass spectrometer and helped CC use mass spectrometer. LL organized control subjects, collected patients, and performed demodex analysis. KW supervised the study, participated in the discussions of each section of experiments, supported this research and reviewed manuscript. All authors read and approved the final manuscript.

## Conflict of Interest Statement

The authors declare that the research was conducted in the absence of any commercial or financial relationships that could be construed as a potential conflict of interest.

## Footnotes

1 www.aao.org/ppp
